# Different Typologies of Gamers Are Associated with Mental Health: Are Students DOOMed?

**DOI:** 10.3390/ijerph192215058

**Published:** 2022-11-16

**Authors:** Turi Reiten Finserås, Børge Sivertsen, Ståle Pallesen, Tony Leino, Rune Aune Mentzoni, Jens Christoffer Skogen

**Affiliations:** 1 Department of Health Promotion, Norwegian Institute of Public Health, 5015 Bergen, Norway; 2 Department of Research and Innovation, Helse Fonna HF, 5525 Haugesund, Norway; 3 Department of Mental Health, Norwegian University of Science and Technology, 7034 Trondheim, Norway; 4 Department of Psychosocial Science, University of Bergen, 5015 Bergen, Norway; 5 Norwegian Competence Center for Gambling and Gaming Research, University of Bergen, 5015 Bergen, Norway; 6 Centre for Evaluation of Public Health Measures, Norwegian Institute of Public Health, 0473 Oslo, Norway; 7 Alcohol and Drug Research Western Norway, Stavanger University Hospital, 4068 Stavanger, Norway

**Keywords:** engaged gamers, Internet Gaming Disorder, mental distress, life satisfaction, young adults

## Abstract

(1) Background: The inclusion of Internet Gaming Disorder in the Diagnostic and Statistical Manual for Mental Disorders (DSM-5) led to a rapid development of assessment instruments based on the suggested diagnosis. However, previous studies suggest that some of the symptoms in the diagnosis reflect engagement in gaming rather than a disorder or addiction. The aim of the present cross-sectional study was to investigate mental health associations with different typologies of gamers. (2) Methods: Data stemmed from a large national survey of students (SHoT2022) that was conducted between February and April 2022 (N = 59,544). Participants were categorized into non-gamers, recreational gamers, engaged gamers, problematic gamers, and addicted gamers. Logistic regression models adjusted for age were analyzed with and without gender-stratification for mental distress and life satisfaction as dependent variables across gaming categories. (3) Results: The proportion reporting case-level mental distress was lower for recreational gamers compared to non-gamers, indicating fewer mental health problems for recreational gamers. However, after stratifying the analysis by gender, female recreational gamers had higher levels of mental distress compared to female non-gamers, reflecting Simpson’s paradox. (4) Conclusions: Future studies investigating mental health and gaming should include a gender perspective.

## 1. Introduction

Gaming disorder was included in the current 11th revision of the *International Classification of Diseases* (ICD-11 [1]), and Internet gaming disorder (IGD) was included under “conditions for further study” in the 5th and current edition of the *Diagnostic and Statistical Manual for Mental Disorders* (DSM-5; [2]). The term IGD will be used in the present study. Still, the research community disagrees as to whether IGD should be considered a mental disorder [3,4], in part because the empirical support is regarded as too weak to determine if IGD is merely a reflection of other underlying problems or represents an independent psychiatric entity [5]. However, some longitudinal studies have suggested that IGD may lead to mental health problems such as depression and anxiety [6,7], indicating that IGD is not just a symptom of other disorders.

Following the inclusion of the proposed IGD diagnosis in DSM-5, several different assessment instruments have been developed, mostly based on the nine suggested DSM-5 criteria [8,9,10,11]. These instruments employ a unidimensional scale and often categorize IGD as endorsing at least five out of nine criteria, in line with the DSM-5. However, it has been proposed that there are different typologies of gamers and that some of the suggested criteria reflect engagement to gaming rather than problematic or addicted gaming [12]. Employing factor analysis, Charlton [12] found that the symptoms of tolerance, mood modification, and cognitive salience loaded high on the engagement factor, while the symptoms of relapse, withdrawal, conflict, and behavioral salience loaded high on what was termed the addiction factor. Charlton and Danforth [13] argue that although engaged gamers may use the same amount of time gaming as addicted gamers, they will not experience the same level of problems caused by their gaming. As such, there is a risk to over-identify addicted gamers if engaged gamers are not considered as a separate category. Later studies have also included a category for borderline addicted gamers (named problematic gamers) and a category for gamers who does not fall into any of the other three categories, named recreational gamers [14].

The prevalence of IGD varies significantly between studies due to differences in assessment and study population [15,16]. The prevalence was reported to be 0.9% in a Norwegian population study [17]. A recent meta-analysis suggests that the prevalence is between 2.0% and 3.1% worldwide for all age groups [18], while a meta-analysis among adolescents found a prevalence of 4.6% [19]. A recent study on a student sample reported a higher prevalence of 5.3% [20]. As such, it seems reasonably established that the prevalence is highest among late adolescents and young adults compared to other age groups [8]. IGD has also consistently been shown to be more prevalent among males than females (e.g., [21,22,23]). This might be because more males play the types of video games that may present a higher risk for developing IGD [24,25]. Furthermore, males on average play more video games than females [26], which could be because more video games are targeted towards males [23], or because females are less encouraged to play [27]. There has also been a concern that IGD increased during the COVID-19 pandemic due to an increase in gaming [28]. Still, studies have reported conflicting results. For example, one study on Japanese adults showed an increase in IGD prevalence [29], a study on Korean adolescents deemed the increase in prevalence to be clinically insignificant [30], and a meta-analysis on the umbrella term internet addiction found no increase in prevalence due to the pandemic [31].

The prevalence has also been shown to be higher when using the DSM-5 IGD-criteria compared to the categorization of gamers, where a distinction is made between addicted and engaged gamers [11]. Using a cut-off of five out of nine criteria according to DSM IGD diagnosis, the prevalence was estimated to be 2.3%. In contrast, the prevalence of addicted gamers was only 1% when the sample was categorized according to Charlton’s typologies of gamers [11,12]. Studies also support a distinction between addicted and engaged gamers in relation to potential mental health and well-being consequences. Some studies based on Charlton’s [12] typology report that problematic and addicted gamers have worse mental health than engaged gamers [7,14,32]. Moreover, a recent study showed that even though addicted and problem gamers had poorer mental health than engaged gamers, engaged gamers had poorer mental health compared to recreational gamers [33]. Inevitably, problems were to be expected, as the participants were assessed with a scale that focuses on problems. However, one study investigating adolescents reported that engaged gamers reported lower psychosocial well-being, but not problems, due to their gaming, while addicted gamers reported both lower psychosocial well-being and problems [34]. In addition, a longitudinal study of the stability of different typologies of gamers showed that none of the gamers transitioned from addicted to engaged gamers, and only two percent transitioned from engaged to addicted over a period of two years, suggesting that being an engaged gamer is not a transitional stage on the way to becoming an addicted gamer for most [7].

In sum, the inclusion of IGD in DSM-5 has led researchers to develop assessment instruments that regard IGD to be a unidimensional construct and often categorize addiction as endorsing at least five out of nine criteria. However, previous studies suggest that some of the criteria better reflect engagement with gaming rather than a disorder or addiction [7,33]. Based on these considerations, the aim of the current study was to investigate mental health associations with different typologies of gamers in a large and recent national student sample of young adults while taking gender into account. In accordance with previous studies, we expected addicted gamers to report lower mental health compared to engaged gamers.

## 2. Materials and Methods

### 2.1. Procedure

The current paper used data from the SHoT2022 study (*Students’ Health and Wellbeing Study*), a large national survey of students enrolled in higher education in Norway. The SHoT2022 is a comprehensive survey of several domains of mental health and lifestyle factors, distributed electronically through a web-based platform. Details of SHOT have been published elsewhere [35]. In short, SHoT2022 was conducted between 8 February and 19 April 2022. The inclusion criteria were being between 18 and 35 years of age, being a full-time student at a college or university, and having a Norwegian citizenship. A total of 169,572 students fulfilled these inclusion criteria (representing all Norwegian full-time students in this age cohort), of whom 59,544 students completed the online questionnaires, yielding a response rate of 35.1%. The reporting in the present paper followed the STROBE guidelines [36].

### 2.2. Instruments

#### 2.2.1. Internet Gaming Disorder (IGD)

The Game Addiction Scale for Adolescents (GASA) is a 7-item scale and was used to measure IGD [37]. Participants indicated their response on a five-point Likert scale (1 = never, 2 = almost never, 3 = sometimes, 4 = often, 5 = very often). Based on the scores, the participants were categorized into four groups of gamers: recreational gamers, engaged gamers, problem gamers, and addicted gamers, according to the categorization of Charlton and Danforth [13]. Participants scoring at least 3/sometimes on the four core criteria (relapse, withdrawal, conflict, and problems) were categorized as addicted gamers. Participants having a score of at least 3/sometimes on two or three of the four core criteria were categorized as problem gamers. Those with a score of 3/sometimes on the three peripheral criteria (salience, tolerance, and mood modification) but scoring 3 or more on no more than one of the core criteria were categorized as engaged gamers. Thus, if participants met the criteria for engagement *and* problem gaming or engagement *and* video game addiction, they were categorized as problem gamers or video-game-addicted gamers, respectively. The participants who confirmed partaking in gaming through the question “*Have you played video games in the last six months?*” but who did not meet the criteria for engaged gamers, problem gaming, or video game addiction were categorized as recreational gamers. The rest of the participants comprised the non-gamers category. The same categorization of gamers has been used in previous studies [21,38]. Cronbach’s alpha of GASA in the current study was 0.83, indicating a very high internal consistency.

#### 2.2.2. Mental distress

Mental distress was assessed by the widely used Hopkins Symptoms Checklist (HSCL-25 [39]), derived from the 90-item Symptom Checklist (SCL-90), which is a screening tool designed to detect symptoms of anxiety and depression. The HSCL-25 consists of 25 items with response categories ranging from “not at all” (1) to “extremely” (4). An investigation of the factor structure based on the SHoT2014 dataset showed that a unidimensional model had the best psychometric properties in the student population and not the original subscales of anxiety and depression [40]. We chose to follow this recommendation in the present study. As recommended in previous publications [39], average scores on the HSCL-25 of ≥1.75/<2.00 and >2.00 were used as cut-off values for identifying moderate and high levels of mental health problems, respectively. Details on development of mental health problems in the SHoT waves were recently published by Knapstad and Sivertsen [41]. Cronbach’s alpha of the HSCL-25 in the current study was 0.94, indicating a very high internal consistency.

#### 2.2.3. Life Satisfaction

The Satisfaction With Life Scale (SWLS; [42]) is a 5-item scale designed to measure global cognitive judgments of one’s life satisfaction (not a measure of either positive or negative affect). In the current study, the SWLS was analyzed in three ways: (1) as a continuous total score (range 5–35), (2) using pre-defined categories (*dissatisfied*: total SWLS score 5–19; *neutral*: total SWLS score 20–25; *satisfied*: total SWLS score 26–35), and (3) dichotomously, using a total SWLS total score of <19 as the cut-off value for indicating poor life satisfaction. The Cronbach’s alpha of the SWLS in the current study was 0.89, indicating a very high internal consistency.

### 2.3. Statistical Analysis

First, the sample characteristics across gaming categories were calculated in terms of mean and standard deviation for continuous variables and in terms of number of observations and proportions for categorical variables. The χ^2^-statistics and Kruskal–Wallis tests were used to assess overall statistical difference between gaming categories. Then, spineplots decomposing the proportion below and above case-level mental distress and the proportion of non-gamers and recreational gamers, overall and stratified by gender, are presented. Problem and addicted gamers were excluded from this particular analysis. Next, the response distribution for scores on HSCL and SWLS were produced across gaming categories stratified for gender, with corresponding boxplots and mean values with 95% confidence intervals. Overall and gender-stratified logistic regression models adjusted for age were analyzed for mental distress and SWLS as dependent variables across gaming categories. In addition, we investigated potential gender moderation by comparing a model with and without the interaction term ‘gaming category × gender’ using likelihood ratio tests. Pairwise comparisons were conducted using Wald tests based on the parameters of the fitted regression models across gaming categories for each gender and across gender for each gaming category. For the latter set of pairwise comparisons, gaming category and gender was entered as an interaction term to allow for category-specific comparisons across gender. Data handling and logistic regression models were conducted using Stata version 15, while descriptive statistics and response distribution with summary statistics were computed using R, version 4.1.1 (R Core [43]) using the following packages: *gtsummary, version 1.6.1* [44], *ggplot2, version 3.3.6* [45], and *ggridges, version 0.5.4* [46]. To include the maximum number of observations in each of the inferential analyses, pairwise deletion was employed.

## 3. Results

### 3.1. Descriptive Statistics

The total number of participants was 58,908, of which 66.7% (*n* = 39 267) were female. Non-gamers consisted of 55.7% of the sample, 37.4% were categorized as recreational gamers, 3.1% as engaged gamers, 3.3% as problem gamers, and 0.5% as addicted gamers. Summary statistics across gaming categories for demographic variables are presented in Table 1. For all included variables, there were significant differences across gaming categories (all *p*-values < 0.001). Overall, non-gamers were more likely to be female and somewhat older. Summary statistics across gaming categories for mental distress and life satisfaction are presented in Table 2. The proportion reporting case-level (>2.0) mental distress increased from engaged gamers to problem and addicted gamers. However, the proportion was lower for recreational gamers compared to non-gamers, indicating fewer mental health problems for recreational gamers. The same pattern was apparent for HSCL mean scores. The proportion reporting good life satisfaction increased from addicted gamers and up to non-gamers.

### 3.2. Associations between Gaming Categories and Mental Distress and Life Satisfaction

**Figure 1:**
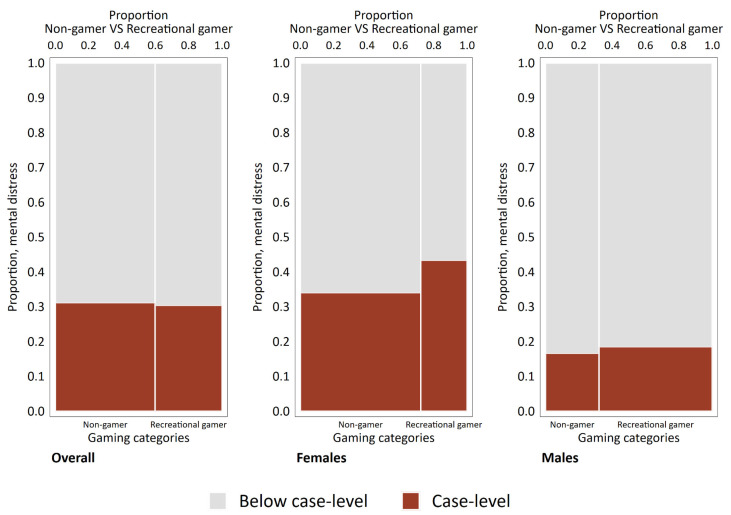
Spineplots showing the distribution of participants in the non-gamer and recreational gamer category both overall and divided by gender across those above and below case-level mental distress.

Figure 1 shows spineplots decomposing the proportion below and above case-level mental distress (y-axis) and the proportion of non-gamers and recreational gamers (x-axis) overall and stratified by gender. Problem and addicted gamers were excluded from this particular analysis. The proportion of case-level mental distress was very similar for non-gamers compared to recreational gamers. The spineplots stratified by gender, however, showed that a higher proportion of female recreational gamers had case-level mental distress compared to female non-gamers. A lower proportion of male participants had case-level mental distress compared to females, and the proportion of mental distress when comparing non-gamers and recreational gamers was more similar for males compared to the pattern observed among females. Even so, when stratified for gender, the proportion of mental distress was higher among recreational gamers than among non-gamers for both females and males.

**Figure 2:**
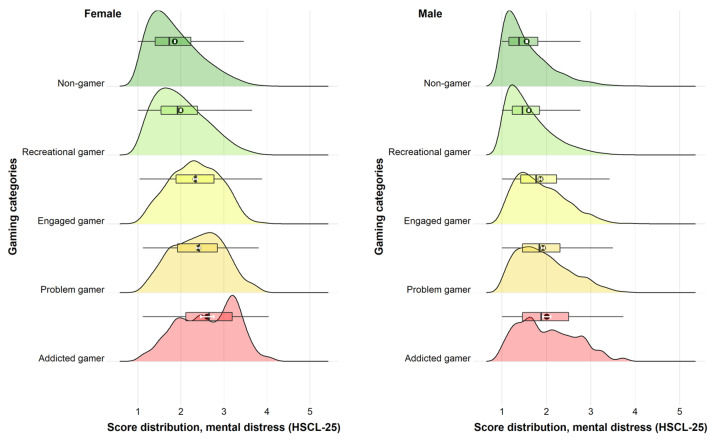
Association between gaming categories and mental distress. Ridgeline plots. Density distributions and corresponding boxplots (with outliers truncated). Black dot indicates mean estimate and white horizontal error bar indicates 95% confidence interval of the mean estimate.

**Figure 3:**
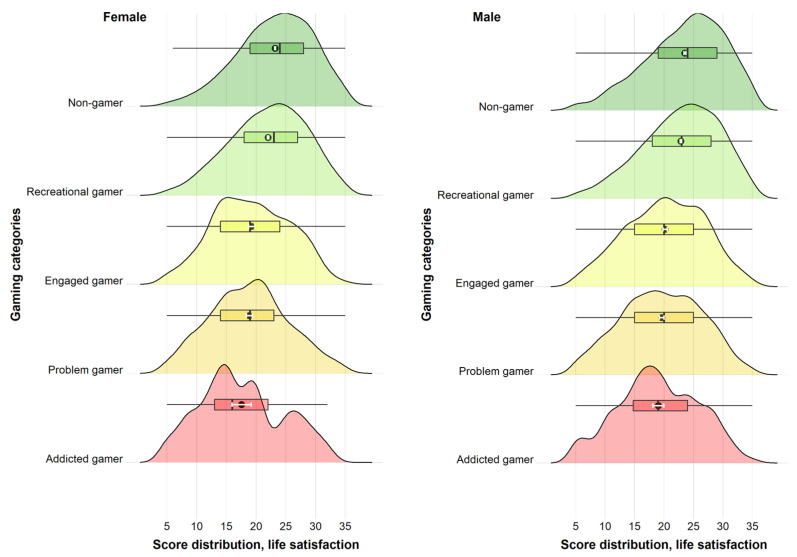
Association between gaming categories and life satisfaction. Ridgeline plots. Density distributions and corresponding boxplots (with outliers truncated). Black dot indicates mean estimate and white horizontal error bar indicates 95% confidence interval of the mean estimate.

Across all dependent variables, we found support for a gender × gaming category interaction (*p*-values ranging from *p* < 0.001 to *p* = 0.002). Therefore, all the main analyses were stratified by gender. The reference category for all analysis was non-gamer. For both females and males, there were monotonous associations with mental distress and life satisfaction across gaming categories (see Figure 2 and Figure 3). For mental distress, the association was positive across gaming categories for both genders, while it was negative for life satisfaction. There was a general tendency for a stronger association for females compared to males across gaming categories in relation to both outcomes. This tendency was most pronounced when looking at recreational, engaged, and problem gamers for mental distress, and when looking at engaged and problem gamers for life satisfaction.

**Figure 4:**
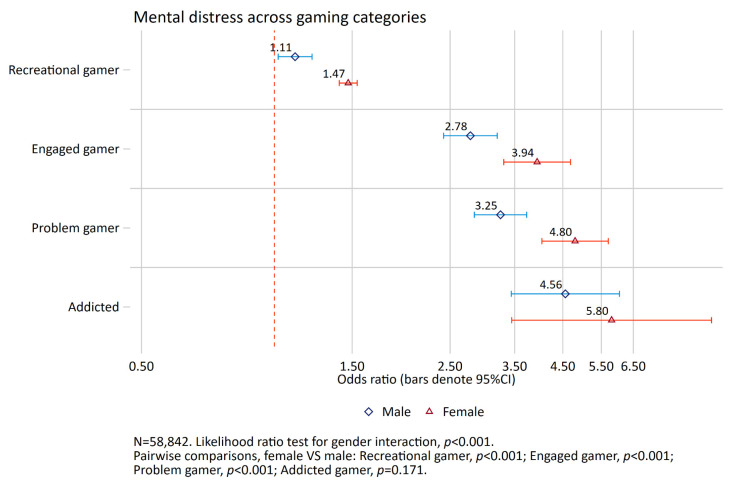
Association between gaming categories and mental distress. Results from logistic regression analysis. Non-gamer as reference group. Adjusted for age, relationship status and living arrangements. The red dashed vertical line indicates odds ratio of 1 (no association).

For mental distress, the odds ratio for problem gamers was 3.25 for males compared to 4.80 for females, while the corresponding estimates for addicted gamers were 4.56 and 5.80 (see Figure 4).

**Figure 5:**
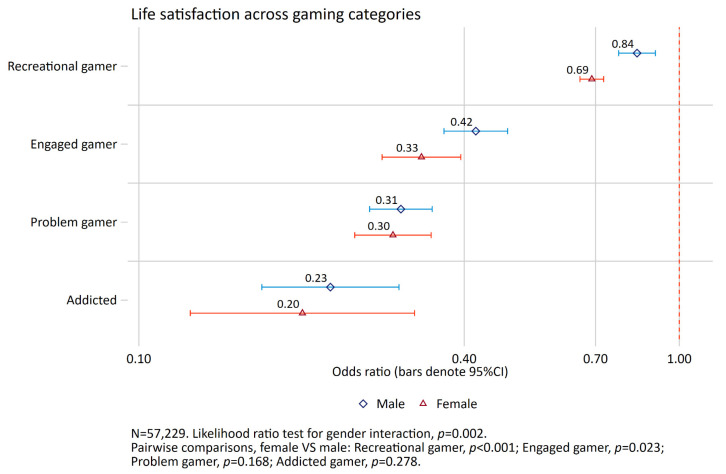
Association between gaming categories and life satisfaction. Results from logistic regression analysis. Non-gamer as reference group. Adjusted for age, relationship status and living arrangements. The red dashed vertical line indicates odds ratio of 1 (no association).

For higher life satisfaction, the odds ratio for problem gamers was 0.31 for males compared to 0.30 for females, while the corresponding estimates for addicted gamers were 0.23 and 0.20 (Figure 5).

Females had higher scores on mental distress in the engaged gamers category compared to the recreational gamer category (*p* < 0.001). There were no significant differences between the other categories. Males had higher scores on mental distress in the engaged gamer category compared to recreational gamers (*p* < 0.001) and in the addicted gamer category compared to the engaged gamers (*p* < 0.001) and problem gamers (*p* < 0.05). Females had higher scores on life satisfaction in the recreational gamer category compared to the engaged gamer category (*p* < 0.001) and in the engaged gamer category compared to addicted gamers (*p* < 0.05). There were no significant differences between the other categories among females. Males had higher scores on life satisfaction in the recreational gamer category compared to engaged gamers (*p* < 0.001) and in the engaged gamer category compared to the problem gamers (*p* < 0.001) and addicted gamers (*p* < 0.001).

## 4. Discussion

The current study investigated different typologies of gamers and their association with mental distress and life satisfaction in a national student sample of young adults. At first sight, the results indicated that recreational gamers reported slightly lower mental distress scores than non-gamers. However, after stratifying the analysis by gender, the opposite was true for females. The results also showed that male addicted gamers had more mental distress and lower life satisfaction than engaged gamers.

The prevalence of addicted gamers was 0.5% in the current study. This is lower compared to other studies [11,18], including a recent population study from Norway [17] and a study of students [20]. However, compared to the Norwegian population study, the current study used a different sample, had a different operationalization of addiction, and assessment took place at different time points (before and after the shutdown due to the COVID-19 pandemic), which might explain the differences in prevalence. The Ohayon and Roberts [20] study of students also included “internet-use” in their definition of IGD, which might have elevated the prevalence estimates. In addition, the prevalence of IGD in Europe has consistently been lower than other continents [18,19], including North America, where the Ohayon and Roberts [20] study was conducted. In comparison, a recent Norwegian study of 19-year-olds employed the similar categorization of gamers and found a prevalence of 1.0%. [11], which is higher than the estimates from the present study. This might be due to the fact that the sample in the current study was slightly older, as previous studies have shown that prevalence are lower in older samples [47]. In addition, we would expect the prevalence to be lower in a student sample, as problematic and addicted individuals might have a higher risk of dropping out of their studies or be more likely to never enroll in higher education.

The present study found that male addicted gamers had 4.6 times higher odds to experience mental distress than male non-gamers, and female addicted gamers had 5.8 times higher odds to experience mental distress compared to female non-gamers. The average level of mental distress in the student sample in the present study is unusually high compared to previous samples [41]. This emphasizes the severity of the symptoms reported by the addicted gamers, as they are heightened compared to the average levels of this student sample. Consistent with previous studies, males in the addicted gamers group experienced more mental distress and lower life satisfaction than engaged gamers [7,14,32,34]. However, for female gamers this only held true for life satisfaction.

The present findings also show that recreational gamers reported more mental distress than non-gamers. The fact that most previous studies (e.g., [17,33]) either combine non-gamers and recreational gamers in one single category or exclude non-gamers from the analysis hampers comparison of this result. Moreover, this finding only appeared after stratifying the analysis by gender, something that might be explained by Simpson’s paradox [48,49]. Simpson’s paradox states that the results from a sample as a whole may be reversed when dividing it into subgroups. As such, the overall analysis masked the difference between non-gamer and recreational gamers on mental distress when taking gender into account (see Figure 1). This highlights the importance of the gender perspective in research concerning mental health and gaming, as females’ health compared to males could be differently impacted by gaming involvement and problems.

There were fewer females in all gaming categorizes. This is consistent with previous studies showing that males spend more time on gaming than females [26] and that IGD is more prevalent in males (e.g., [21,22,23]). Still, females experienced significantly higher mental distress and lower life satisfaction in the recreational and engaged gamers categories compared to male gamers in the respective categories. Twenge and Martin [50] found that heavy female gamers had a higher relative risk of experiencing mental distress than heavy male gamers. Indeed, female gamers have reported that they experience a higher degree of discrimination and harassment when they reveal their gender while gaming [51]. Females might be viewed as having lower skill levels than their male counterparts, as gaming has been viewed as a predominantly male activity. The harassment females experience might explain why they also experience more mental distress compared to male gamers, especially in the recreational gamers category. On the other hand, some studies on adolescents have shown that non-gamers have worse health than recreational gamers [52,53], which may suggest that males who completely refrain from gaming are a marginalized group.

### Strengths and Limitations

There are some limitations to the current study that should be mentioned. The cross-sectional design puts limits in terms of conclusions about directionality. The study is vulnerable for known biases like recall bias, social desirability bias, and the common method bias. The results of the study may not be generalizable to the general population, as the participants were students and young adults. In addition, the response rate in the current study was relatively modest (35.1%), which might have impacted the prevalence estimates, as non-participation has been linked to poorer mental health [54]. Still, low response rates likely influence estimates of relationships between study variables to a lesser degree [54,55]. A notable strength of the study is the recency of the data collection. In addition, the large sample allowed for categorization into typologies of gamers stratified by gender while keeping the statistical power at a high level, which is rare in studies on this topic, as there normally are few female gamers in the addicted category. This also allowed for the results that showed differences in mental distress and life satisfaction between female non-gamers and female recreational gamers.

## 5. Conclusions

The results of this study showed a lower prevalence of addicted gamers in a student sample than in other populations. Furthermore, engaged, problem, and addicted gamers showed significantly more mental distress and less life satisfaction than recreational and non-gamers in both males and females. As reported by previous studies, there were significant differences between engaged gamers and addicted gamers, where the latter experienced more mental distress and less life satisfaction. Females showed significantly more mental distress in the recreational gamer category compared to the non-gamer category and tended to experience higher distress in the other categorizes compared to males. This might reflect the high degree of discrimination, stigma, and harassment that females continue to report when gaming. Future studies should continue to explore the different typologies of gamers and include non-gamers as a category. There is especially a need for more studies focusing on gender perspectives and female gamers in particular.

## Figures and Tables

**Table 1:** Descriptive statistics.

Characteristic	Non-Gamer, *N* = 32,811 ^1^ (55.7%)	Recreational Gamer, *N* = 22,013 ^1^ (37.4%)	Engaged Gamer, *N* = 1838 ^1^ (3.1%)	Problem Gamer, *N* = 1954 ^1^ (3.3%)	Addicted Gamer, *N* = 294 ^1^ (0.5%)	*p*-Value ^2^
Gender						<0.001
Female	27,381 (70%)	10,542 (27%)	610 (1.6%)	657 (1.7%)	78 (0.2%)	
Male	5430 (28%)	11,471 (58%)	1228 (6.3%)	1297 (6.6%)	216 (1.1%)	
Age categories						<0.001
18–20 yrs	4691 (56%)	3109 (37%)	273 (3.3%)	272 (3.2%)	39 (0.5%)	
21–22 yrs	8859 (55%)	6065 (38%)	488 (3.0%)	548 (3.4%)	76 (0.5%)	
23–25 yrs	9158 (53%)	6858 (40%)	638 (3.7%)	604 (3.5%)	89 (0.5%)	
26–28 yrs	3205 (50%)	2614 (41%)	250 (3.9%)	250 (3.9%)	46 (0.7%)	
29–35 yrs	2867 (56%)	1884 (37%)	150 (2.9%)	186 (3.6%)	36 (0.7%)	
36+ yrs	4031 (71%)	1481 (26%)	39 (0.7%)	94 (1.7%)	8 (0.1%)	
Relationship status						<0.001
Single	15,710 (56%)	10,616 (38%)	957 (3.4%)	838 (3.0%)	107 (0.4%)	
Boyfriend/girlfriend	6831 (55%)	4691 (38%)	394 (3.2%)	445 (3.6%)	72 (0.6%)	
Cohabitant/married/registered partner	10,194 (56%)	6654 (37%)	483 (2.7%)	666 (3.7%)	114 (0.6%)	
Living arrangements						<0.001
Alone	6730 (57%)	4363 (37%)	402 (3.4%)	373 (3.1%)	38 (0.3%)	
With partner	10,337 (56%)	6719 (37%)	498 (2.7%)	662 (3.6%)	131 (0.7%)	
With friends/others in a collective	13,343 (56%)	8958 (38%)	713 (3.0%)	673 (2.8%)	88 (0.4%)	
With parents	2199 (48%)	1886 (41%)	221 (4.8%)	241 (5.3%)	37 (0.8%)	

^1^*n* (%); mean (*SD*). ^2^ Pearson’s chi-squared test; Kruskal–Wallis rank sum test. *Note:* decimal points are only shown for numbers less than ten.

**Table 2:** Descriptive statistics for mental distress and life satisfaction across gaming categories.

Characteristic	Non-Gamer,	Recreational Gamer,	Engaged Gamer,	Problem Gamer,	Addicted Gamer,	*p*-Value ^2^
	*N* = 32,811 ^1^	*N* = 22,013 ^1^	*N* = 1838 ^1^	*N* = 1954 ^1^	*N* = 294 ^1^	
HSCL ^3^, mean score	1.81 (0.58)	1.79 (0.58)	2.02 (0.60)	2.08 (0.63)	2.17 (0.71)	<0.001
Mental distress						<0.001
Below case-level	22,544 (69%)	15,301 (70%)	971 (53%)	983 (50%)	136 (46%)	
Case-level (>2.0)	10,221 (31%)	6694 (30%)	866 (47%)	970 (50%)	158 (54%)	
Life satisfaction, score	23 (6)	22 (6)	20 (7)	19 (7)	19 (7)	<0.001
Life satisfaction						<0.001
Poor life satisfaction	8593 (27%)	6738 (31%)	847 (47%)	968 (51%)	161 (57%)	
Good life satisfaction (ref.)	23,236 (73%)	14,681 (69%)	952 (53%)	933 (49%)	122 (43%)	

^1^*n* (%); mean (*SD*). Column percentages. ^2^ Pearson’s chi-squared test; Kruskal–Wallis rank sum test. ^3^ Hopkins Symptoms Checklist.

## Data Availability

The datasets presented in this article are not readily available because of privacy regulations from the Norwegian Regional Committees for Medical and Health Research Ethics (REC). Approval from REC (https://helseforskning.etikkom.no, accessed on 17 October 2022) is a pre-requirement. Guidelines for access to SHoT data are found at: https://www.fhi.no/en/more/access-to-data (accessed on 17 October 2022). Requests to access the datasets should be directed to borge.sivertsen@fhi.no.
